# Care of the Insane in the United States

**Published:** 1887-10

**Authors:** Judson B. Andrews

**Affiliations:** Buffalo, N. Y., President of Section in Psychological Medicine and Nervous Diseases


### *THE DISTRIBUTION AND CARE OF THE INSANE IN THE UNITED STATES.*

By JUDSON B. ANDREWS, M. D., Buffalo, N. Y.,
President of Section in Psychological Medicine and Nervous Diseases.

After delivering an eloquent eulogy on Dr. John P. Gray, who was the first president of this section, he said: The amount of insanity bears a close relation to the duration of the social and governmental life of the people. Dividing the country into two great belts of north and south, there is an almost regular proportionate decrease of lunacy as we leave the older settled parts of the country along the Atlantic coast, until we reach the extreme western slope.

The New England states lead with one insane person to every 359 inhabitants. This decreases until we reach the newer states and territories, with one insane person to every 1,263 inhabitants. In the seaboard states of the southern belt, we have one to every 610 inhabitants, and the extreme southern states with but one to every 935 of the population. This emphasizes the statement that the pioneers of our new settlements are the most hard and vigorous citizens, and that the feeble and dependent are left in their former homes to enjoy the comforts of the hospitals and asylums, which are the special growth of the old civilization.

New York is the first Irish city in the world, and Berlin and Hamburg are the only cities which contain as many Germans as our own metropolis. We have among the negroes in the United States one insane person to every 1,097 of inhabitants. In the negro race, the proportionate increase of insanity is far greater than in any other division of the population. From 1870 to

1880, there was an increase in the census of the colored race of thirty-four and eighty-five-hundredths per cent., while for the same period there was an increase of twenty-five and eight-tenths per cent. of the insane. The large multiplication has occurred since the emancipation from slavery and the consequent change in condition and life. The causes are briefly told—enlarged freedom, too often ending in license; excessive use of stimulants; excitement of the emotions, already unduly developed; the unaccustomed strife for means of subsistence; educational strain and poverty. Among the Chinese and aborigines there has been but a small increase of insanity. There is among them less of the refinements of civilization, less competition and struggle for place, power or wealth, and, as a consequence, less tendency to mental deterioration.

Dr. Andrews presented a list of state institutions in the United States, number of patients in each, and also the number of medical officers attached to each institution. One hundred and twenty-one asylums were represented, fifteen of these having been built since 1880. In 1880, there was 39,093 patients in these asylums. In 1886, the number had increased to 61,411, an annual increase of nine per cent. At the present rate of increase, before the end of the decennial period, we shall have 75,000 in our asylums.

Dr. Andrews next referred to the diversity in lunacy laws in the various states of the union, comparing them with the general governmental laws of Great Britain. Special accommodation for the criminal class, a highly desirable innovation, has been entered upon by two states only—New York and Illinois.

With the exception of Delaware and Vermont, every state in the union has adopted the state system of caring for the insane, and New York was the first state to erect separate asylums for the chronic insane. Asylum architecture has undergone material changes during the past twenty years, the plan now most generally adopted being that of building detached blocks of structures in different parts of the asylum grounds, within easy reach of the administration building, these to accommodate the feeble and helpless, the epileptics and acutely maniacal patients. In many portions of the country, buildings separate and complete are joined by fire-proof corridors.

The president next outlined some of the important changes occurring in the interior management of asylums at the present time; among them, the introduction of electricity for lighting purposes, of direct radiation for heating purposes, and of natural ventilation as taking the place of the blower-fans, much in vogue in former years.

A higher medical standard is to be noted in the asylums of the United States. The medical officers are in very few cases appointed through political favoritism, and in the state of New York the civil service requirements insure the sound medical equipment of assistant physicians. Oöphorectomy is now recognized as a legitimate mode of treatment, and castration in appropriate cases has, at present, some able advocates; electricity is now being used with more intelligent knowledge of its power. Its hand-maid, massage, less powerful and less mysterious, but not less practical, has gained a position of prominence in the treatment of insanity in many institutions. The experiments in mesmerism, mind-reading and the faith-cure have led to a closer investigation in the relation between mind and body, with a result of finding, in expectant attention, a valuable and legitimate help in the treatment of mental diseases.

